# Development and application of SNP chips in goat breeding: a mini review

**DOI:** 10.3389/fvets.2026.1820954

**Published:** 2026-07-29

**Authors:** Ting-Chieh Kang, Hisn-Hung Lin, Kai-Fei Tseng, Yu-Hsin Chen, Ya-Chun Liu

**Affiliations:** 1 Southern Branch, Taiwan Livestock Research Institute, Ministry of Agriculture, Pingtung, Taiwan; 2 Taiwan Livestock Research Institute, Tainan, Taiwan

**Keywords:** genetic diversity, genome-wide association study, genomic selection, goat, SNP chip

## Abstract

Goats (*Capra hircus*) are among the world’s most important livestock, providing milk, meat, and fiber across diverse agro-ecological zones. Traditional breeding relying on pedigree-based estimated breeding values (EBVs) has driven steady genetic progress but is constrained by long generation intervals and limited accuracy for sex-limited or difficult-to-measure traits. High-throughput single nucleotide polymorphism (SNP) chips and genomic selection (GS) have transformed goat breeding by enabling early, accurate selection independent of phenotypic records. This review synthesizes the development of goat SNP chip platforms from the foundational 52 K GoatSNP50 BeadChip through high-density solid-phase arrays and low-cost liquid-phase capture panels, with emphasis on their relative performance, cost-effectiveness, imputation potential, and suitability for different breeding systems. In addition to genomic selection (GS), genome-wide association studies (GWAS), and genetic diversity assessment, we also discuss candidate-gene selection and marker-assisted selection (MAS) as practical intermediate approaches that remain relevant in many goat breeding programs. GS has achieved genomic estimated breeding value (GEBV) prediction accuracies of 0.35–0.79 for key production traits across multiple countries and breeds. GWAS has identified candidate genes for milk composition (DGAT1, CSN1S1), growth (PLAG1, HMGA2), reproduction (BMPR1B, GDF9), and fiber quality (KRT, KRTAP families). We compare GS with traditional BLUP-based approaches, assess economic benefits, and discuss key challenges including reference population construction, genotype imputation, inbreeding management via Optimum Contribution Selection (OCS), and multi-omics integration. Future directions include customized chip design, AI-assisted genomic prediction, climate adaptation breeding, and CRISPR/Cas9 gene editing for precision improvement.

## 1 Introduction

Goats (*Capra hircus*) were among the first domesticated livestock and play a vital role in the rural agricultural economies of developing regions and resource-limited production systems, serving as a critical source of food security for smallholder farmers worldwide. Their exceptional adaptability to diverse environments from arid deserts to high-altitude mountains allows them to provide a vital source of high-quality protein (milk and meat) and natural fibers (cashmere and mohair) for hundreds of millions of people worldwide (1, 2). With the increasing global demand for animal protein and the challenges posed by climate change, accelerating the genetic improvement of goats to enhance production efficiency, disease resistance, and environmental adaptability has become a critical research priority in animal science (3).

Traditional breeding methods have primarily relied on phenotypic selection and pedigree records to estimate breeding values (EBVs) for selecting superior individuals. While this approach has achieved considerable success, its accuracy is fundamentally constrained by the heritability of target traits and the completeness of pedigree information. For traits expressed only in one sex (e.g., lactation) or requiring post-slaughter measurement (e.g., carcass traits), the selection cycle is both long and costly (3, 4). Furthermore, classical BLUP-based evaluation cannot distinguish Mendelian sampling variation among full siblings, limiting the resolution of within-family selection.

To overcome the inherent limitations of pedigree-based methods, the caprine research community transitioned toward high-throughput molecular tools, a shift made possible by the publication of the first high-quality reference genome assemblies. The sequencing of the goat reference genome (ARS1 and subsequent assemblies) provided the chromosomal scaffold necessary for high-density marker development and accurate SNP localization across the genome (5). These genomic resources laid the foundation not only for genomic selection (GS) but also for genome-wide association studies (GWAS) and population-level genetic diversity assessment, three complementary applications that together define modern goat genomics. Alongside GS, GWAS and genetic diversity assessment represent equally foundational pillars of modern goat genomics. GWAS identifies SNP markers statistically associated with complex production traits across the genome, thereby clarifying their genetic architecture and providing candidate functional loci that inform both marker-assisted selection and GS model construction (3, 4). Genetic diversity assessment, in turn, provides the population genetic foundation upon which all genomic improvement strategies depend. Two parameters are particularly critical: effective population size (Ne) and linkage disequilibrium (LD). In goats, Ne has declined markedly over domestication history, reducing effective recombination and increasing genomic relationship among individuals (6). LD in goats decays more rapidly than in cattle, typically reaching r^2^ < 0.3 within 200–500 kb, necessitating higher marker densities for accurate GS and GWAS (6, 7). These parameters determine the minimum SNP density required for reliable genomic prediction, the minimum reference population size needed to sustain accuracy, and the risk of inbreeding depression associated with uncontrolled selection in small populations (3, 4, 6). All three applications—GS, GWAS, and genetic diversity assessment—are therefore mutually reinforcing: GWAS findings can up-weight functional loci in Bayesian GS models, while Ne and LD analyses guide SNP panel design and breed conservation strategy (3, 4). The concept of genomic selection (GS) was introduced by (8), with the core principle of using genome-wide markers to predict an individual’s genomic estimated breeding value (GEBV) regardless of whether a phenotypic record exists. GS is particularly advantageous for early selection, traits expressed late in life, or those that are difficult or expensive to measure. By simultaneously increasing selection accuracy and shortening the generation interval, GS can dramatically accelerate annual genetic gain (9, 10). Following remarkable success in the dairy cattle industry, GS has progressively been extended to sheep, pigs, poultry, and goats (4).

However, the transition from conventional phenotypic selection to genome-wide selection has not occurred uniformly across regions or production systems. In many developing countries and smallholder goat systems, candidate-gene selection and MAS remain more accessible than whole-genome GS because they require lower genotyping investment and can be applied to traits influenced by major genes. For example, reproductive loci in the BMP signaling pathway, including BMPR1B, GDF9, and BMP15, have been widely considered for litter size, ovulation rate, and related fertility traits. These approaches provide a practical bridge between traditional selection and GS, although they capture only a limited fraction of the genetic variance for highly polygenic traits.

Despite its potential, the widespread adoption of GS in goat breeding faces practical barriers including the high cost of genotyping at scale, the challenge of constructing sufficiently large and representative reference populations, and the genetic complexity of many economically important traits. This review addresses these challenges by providing a comprehensive synthesis of: (1) the development and technical characteristics of goat SNP chip platforms; (2) the applications and outcomes of GS, GWAS, and genetic diversity assessment; (3) a critical comparison of GS versus traditional breeding; and (4) the economic rationale and emerging future directions including multi-omics integration, AI-assisted prediction, and gene editing for goat genomic improvement. By synthesizing these advances, we aim to provide researchers and breeders with a systematic reference that identifies both the current state-of-the-art and the key gaps that must be bridged to translate genomic tools into routine breeding practice.

## 2 Technical principles and development of SNP chips

The development of SNP chips represents a milestone in goat genomics, providing essential tools for GS and GWAS applications. The evolution from medium-density and high-density solid-phase arrays to flexible liquid-phase capture technologies reflects continuous technological advancement and the progressive reduction in per-sample genotyping costs (Table 1).

**Table 1:** Development history of goat SNP chip technology.

Chip name	Year	SNP count	Developer/consortium	Type	Primary application and key reference(s)
GoatSNP50 (52 K)	2014	53,347	IGGC / Illumina	Solid-phase	GWAS, GS, genetic diversity, population structure (11–13)
Cashmere 66 K	2017	~66,000	Chinese research teams	Solid-phase (custom)	GS for cashmere yield traits; targeted breed-specific application (14)
High-density 70 K+	2022+	>70,000	Multiple consortia	Solid-phase	Improved cross-breed GS accuracy; fine-scale QTL mapping (15)
Liquid-phase 25 K	2023	~25,000	Chinese dairy goat teams	Liquid-phase capture	Low-cost GS; parentage testing; commercial dairy goat programs (16)
Liquid-phase 54 K	2023	~54,000	Chinese dairy goat teams	Liquid-phase capture	High-accuracy GS at reduced cost; scalable deployment for breeding programs (17)

From a practical breeding perspective, no single SNP platform can be considered universally superior. The optimal platform depends on the breeding objective, target breed, linkage disequilibrium structure, reference population size, required marker density, and genotyping budget. The GoatSNP50 BeadChip remains the most broadly validated platform and is therefore particularly valuable for genetic diversity studies, across-study comparisons, and routine GWAS in populations where 50 K density provides adequate genome coverage. In contrast, high-density and breed-customized arrays are more appropriate for fine-scale QTL mapping, cross-breed prediction, and imputation to denser marker sets, but their higher cost may limit routine use in small breeding programs.

Liquid-phase capture panels represent a different trade-off. Their main advantage is not necessarily higher marker density, but flexibility and scalability: probe sets can be updated, breed-specific markers can be incorporated, and per-sample costs may be reduced when large numbers of animals are genotyped. Therefore, recent 25 K and 54 K liquid-phase panels for dairy goats may be especially useful for commercial GS, parentage verification, and low-cost population screening, provided that the selected markers are informative for the target population and supported by an appropriate reference panel.

### 2.1 Birth and application of the 52 K SNP Chip

In the early stages of goat genomics, researchers relied primarily on microsatellite markers for genetic analysis, but their limited number prevented whole-genome inference. To address this, the International Goat Genome Consortium (IGGC) led the development of the first commercial medium-density goat SNP chip, the Illumina GoatSNP50 BeadChip published in 2014 (11). The array contains 53,347 SNPs selected from whole-genome resequencing data across diverse worldwide breeds, with validation confirming broad polymorphism across major dairy, meat, and fiber breeds. This chip rapidly became the standard tool for studying genetic diversity and population structure (12, 13), as well as GWAS (11) and genomic selection (4) in goats globally, and remains widely used today due to its extensive validation and the large volume of comparable published data accumulated across worldwide breeds.

### 2.2 High-density and custom chips

As GS applications expanded, researchers recognized that higher marker density could improve prediction accuracy, particularly for cross-breed or admixed-population analyses where shorter haplotype blocks require denser coverage (3, 4). This motivated the development of higher-density or breed-customized arrays. A 66 K chip specifically designed for cashmere yield traits in Cashmere goats was developed by integrating genome-wide target enrichment data (14), providing superior coverage of genomic regions relevant to fiber production. More recently, panels exceeding 70,000 SNPs have been reported, enabling finer-scale QTL mapping and improved imputation accuracy for less-common variants (15).

### 2.3 Advances in liquid-phase capture chips

To overcome the high cost and fixed marker composition of traditional solid-phase arrays, liquid-phase capture technology based on targeted sequence capture followed by high-throughput sequencing has emerged as a cost-effective alternative. Also termed genotyping-by-sequencing (GBS), this approach uses designed oligonucleotide probes to selectively capture and sequence target genomic regions, enabling flexible and scalable genotyping. Recent studies have successfully developed 25 K and 54 K liquid-phase SNP panels for dairy goats, with performance comparable to solid-phase chips of equivalent density but at substantially lower per-sample cost (16, 17). These liquid-phase panels greatly enhance the commercial feasibility of GS in smaller breeding programs.

### 2.4 Genotype imputation: bridging density gaps

Genotype imputation, the statistical inference of unobserved genotypes using haplotype reference panels, represents a critical bridge between low-cost, low-density genotyping and the high-density marker sets required for accurate GS. By genotyping selection candidates with low-density panels (e.g., 5 K–15 K SNPs) and imputing to 50 K + density using a reference panel of high-density genotyped individuals, breeding programs can substantially reduce per-animal genotyping costs while maintaining prediction accuracy (18). The accuracy of imputation depends on marker density in the low-density panel, linkage disequilibrium (LD) structure in the population, and reference panel size (18). In goats, LD decays more rapidly than in cattle, necessitating careful selection of tag SNPs to ensure robust imputation (6). The development of population-specific imputation panels optimized for each target breed’s LD architecture (11), alongside advances in software tools such as Beagle and FImpute (18), is expected to further democratize genomic evaluation in resource-limited settings.

Consequently, imputation accuracy should not be interpreted as a fixed property of a chip alone. A low-density or liquid-phase panel may perform well in one breed but poorly in another if the reference population does not capture the relevant haplotypes. In goats, where LD often decays faster than in cattle and many breeds have small effective population sizes, imputation is expected to be most reliable when reference animals are closely related to selection candidates, when marker spacing is optimized for the target breed, and when rare or breed-specific haplotypes are adequately represented. Thus, high-density arrays generally provide the strongest basis for imputation reference panels, whereas low-density and liquid-phase panels are most cost-effective when used together with well-designed breed-specific reference resources.

## 3 Applications of SNP chips in goat breeding

The widespread adoption of SNP chips has driven three complementary applications in goat breeding: genomic selection (GS), genome-wide association studies (GWAS), and genetic diversity assessment. These approaches are mutually reinforcing: GWAS discoveries can inform GS model construction, while population genomics findings guide conservation and breed management decisions.

### 3.1 Genomic selection (GS)

The core of GS is to use genome-wide SNP markers to predict an individual’s GEBV, enabling early and accurate selection before phenotypic expression. Three broad statistical frameworks underpin GS in goats: (1) Genomic Best Linear Unbiased Prediction (GBLUP), which replaces the pedigree-based relationship matrix with a genomic relationship matrix (G-matrix) constructed from SNP data; (2) single-step GBLUP (ssGBLUP), which simultaneously incorporates phenotyped-and-genotyped, phenotyped-only, and genotyped-only individuals in a unified model, maximizing the use of all available information; and (3) Bayesian methods (e.g., BayesB, BayesC), which apply variable selection across markers and are particularly powerful when a subset of loci explains a large proportion of trait variance, such as the CSN1S1 locus in dairy goats. Teissier et al. (10) demonstrated that weighted ssGBLUP, which up-weights markers in genomic regions with large effects significantly improved accuracy for milk protein content in French Alpine and Saanen goats compared to standard GBLUP, highlighting the importance of model choice for traits influenced by major genes. Since France pioneered national dairy goat GS, multiple countries have implemented GS programs across different breeds and production systems (Table 2). More recently, studies have begun integrating GWAS prior information into GS models, improving prediction accuracy for cashmere traits in Inner Mongolia Cashmere goats (19).

**Table 2:** Examples of genomic selection (GS) applications in goats worldwide.

Country	Breed(s)	Model	Target trait(s)	Accuracy (r)	Key finding and reference(s)
France	Alpine, Saanen	GBLUP / ssGBLUP	Milk yield, protein, fat	0.55–0.75	First national dairy goat GS scheme; ssGBLUP outperformed GBLUP for CSN1S1-influenced traits (10, 21)
UK	Mixed dairy breeds	GBLUP	Milk yield, conformation	0.50–0.68	Multi-breed reference population improved prediction accuracy for under-represented breeds (9)
Canada	Alpine, Saanen	ssGBLUP	Milk, fat, protein yield	0.61–0.79	Single-step integration of historical pedigree data increased accuracy by ~12% over multi-step GBLUP (22, 47)
New Zealand	Dairy goats	GBLUP	Milk, fat, protein, SCS	0.45–0.70	Genomic EBVs released publicly; SCS heritability confirmed lower than cattle (48)
China	Inner Mongolia Cashmere	GBLUP + GWAS priors	Cashmere yield, fibre diameter	0.40–0.65	Integrating GWAS prior information into GS model improved accuracy for cashmere traits (19)
South Africa	Indigenous breeds	GBLUP	Carcass traits	0.35–0.58	Genomic regions for carcass quality identified; small reference population limits accuracy (2, 27)

The variation in reported prediction accuracies among countries and breeds should be interpreted critically. Higher accuracies reported in Canadian Alpine and Saanen dairy goats are likely attributable to several favorable conditions, including larger and better connected pedigree structures, long-term milk recording, relatively standardized phenotypes, and the use of ssGBLUP models that integrate historical pedigree, phenotype, and genotype information. By contrast, the lower accuracies reported for South African indigenous goats may reflect smaller reference populations, more heterogeneous breed composition, weaker relationships between reference and validation animals, and the greater difficulty of recording carcass traits consistently under diverse production environments.

Marker density is only one component of prediction accuracy. In practice, the size and representativeness of the reference population, trait heritability, extent of LD between markers and QTL, phenotype quality, breed structure, and statistical model often have greater influence than marker number alone. For traits controlled partly by major genes, weighted ssGBLUP or Bayesian models that allow unequal marker effects may outperform standard GBLUP. For highly polygenic traits, increasing reference population size and improving phenotype quality may be more beneficial than simply increasing SNP density. These differences explain why GS appears more successful in some dairy goat systems than in smaller, less structured meat or indigenous populations.

These studies consistently demonstrate that GS enhances genetic progress for traits such as milk yield, cashmere production, meat quality, and fertility. Its advantage is particularly pronounced in shortening generation intervals and enabling evaluation of traits that are difficult, expensive, or impossible to measure directly-such as carcass traits prior to slaughter or dairy traits in buck progeny.

### 3.2 Genome-wide association study (GWAS)

GWAS aims to identify SNP markers statistically associated with specific traits by testing millions of marker-phenotype associations across the genome (4, 11). This approach localizes quantitative trait loci (QTL) and narrows candidate gene lists, providing a prerequisite for understanding trait genetic architecture and developing functional markers for marker-assisted selection (MAS) (3). Statistical power depends critically on sample size, trait heritability, effect size distribution, and the multiple-testing correction applied; most studies use Bonferroni correction (genome-wide significance threshold *p* < 5 × 10^−8^) or false discovery rate (FDR) control to balance Type I and Type II error rates (4, 20). Through GWAS, researchers have identified a growing catalog of candidate genes for economically important traits in goats (Table 3).

**Table 3:** Selected candidate genes affecting important traits in goats identified via GWAS.

Trait category	Gene/locus	Chr.	Breed/population	Functional relevance and reference(s)
Milk production	DGAT1	14	Saanen, Alpine	Encodes diacylglycerol O-acyltransferase 1; major effect on milk fat percentage and composition (21)
Milk production	CSN1S1, CSN2	6	French dairy breeds	Casein gene cluster; polymorphisms directly affect milk protein composition and cheese-making quality (10, 21)
Milk production	LALBA, LTF	Multiple	Saanen (Canada, China) and UK (mixed dairy)	Lactalbumin and lactoferrin genes; associated with milk yield and somatic cell score variation (22–24)
Growth and body conformation	PLAG1, HMGA2	14, 4	Multiple breeds	Pleiomorphic adenoma gene 1 and high-mobility group AT-hook 2; major regulators of body size and growth rate across livestock species (4, 20)
Growth and body conformation	MSTN, IGF1	2, 17	Meat breeds	Myostatin inhibits muscle growth; IGF1 promotes skeletal muscle hypertrophy; variants linked to improved lean muscle deposition (27, 28)
Growth and body conformation	Zhongwei-specific QTL	Multiple	Zhongwei goat	Novel loci for body length, height, and cannon bone circumference unique to this adapted local breed (20)
Reproduction	BMPR1B (FecB)	6	High-prolificacy breeds	Bone morphogenetic protein receptor 1B; FecB mutation is the most well-characterised litter-size gene in small ruminants (33, 34)
Reproduction	GDF9, BMP15	5, X	Youzhou dark goat	Growth differentiation factor 9 and bone morphogenetic protein 15; regulate ovulation rate and litter size in goats (33)
Fibre quality	KRT, KRTAP genes	Multiple	Cashmere, Angora	Keratin and keratin-associated protein genes; variants linked to cashmere fibre diameter, yield, and crimp (14)
Meat quality	CALD1, ACTA2	Multiple	Karachai, SA breeds	Caldesmon and smooth muscle actin genes; associated with intramuscular fat and muscle fibre composition affecting meat texture (27, 28)

Nevertheless, the robustness of goat GWAS findings varies considerably among studies. Associations reported in large, well-recorded populations and replicated across breeds should be considered more reliable than signals detected in a single small population. Sample size, population stratification, relatedness among animals, phenotype definition, multiple-testing correction, and marker density all influence the probability of false-positive or false-negative findings. Therefore, GWAS results should be evaluated not only by statistical significance but also by biological plausibility, consistency across populations, and independent validation.

Several candidate loci, such as DGAT1 and the casein gene cluster for milk composition, and genes in the BMP pathway for reproductive traits, have stronger biological support because their physiological roles are well established. In contrast, many newly reported loci for growth, conformation, carcass, and adaptation traits remain preliminary until confirmed in independent populations or supported by functional evidence such as expression, protein function, or pathway-level validation. This distinction is important because unvalidated GWAS signals may be useful for hypothesis generation but should be applied cautiously in MAS or GS model weighting.

For milk production traits, DGAT1 on chromosome 14 encodes diacylglycerol O-acyltransferase 1, the final rate-limiting enzyme in triglyceride synthesis; its polymorphisms alter enzymatic activity and thus explain a substantial proportion of variation in milk fat content across multiple goat breeds (21). The casein gene cluster (CSN1S1, CSN2, CSN3) on chromosome 6 encodes the primary structural proteins of the casein micelle, and variants affecting their expression levels and amino acid sequences directly determine milk protein composition, micelle stability, and technological properties critical for cheese-making quality (10, 21). These findings highlight that GWAS not only maps genomic loci but also connects genetic variation to specific metabolic and physiological pathways underlying production traits. Subsequent GWAS in Canadian Alpine and Saanen populations identified additional milk yield and conformation candidates, including LALBA and LTF as key genes associated with milk yield and somatic cell score variation (22). In the UK, a GWAS in mixed-breed dairy goats similarly identified genomic loci associated with both milk yield and body conformation traits (23). More recently, a GWAS in Chinese Saanen goats identified CDC14A, F11, RBPJL, and ZFAND2A as novel candidate genes for milk production performance (24). The genetic architecture of growth and meat production in goats is shaped by at least two major biological axes: the regulation of prenatal and postnatal growth, and the control of post-mortem proteolysis governing meat tenderness. PLAG1 and HMGA2 have been identified as key growth regulators in multiple goat populations (4, 20). At the molecular level, HMGA2 encodes a non-histone chromatin architectural protein that activates PLAG1 expression through chromatin remodeling (25), while PLAG1 directly upregulates IGF2 transcription from the P3 promoter, stimulating fetal and postnatal growth through the conserved HMGA2–PLAG1–IGF2 axis (26). Disruption of any node in this pathway results in intrauterine and postnatal growth retardation across mammalian species, confirming its conserved functional importance (26). Breed-specific GWAS, such as in the Zhongwei goat, have also uncovered novel loci for body structural traits not previously reported in other breeds (20). MSTN and IGF1 remain key candidates for muscle mass and growth rate in meat breeds (27, 28). In the context of meat quality, the calpain–calpastatin system (CAPN1, CAPN2, and their endogenous inhibitor CAST) plays a central role in post-mortem proteolysis: CAPN1 (*μ*-calpain) degrades key myofibrillar structural proteins including titin, desmin, and nebulin during the aging process, with CAST modulating the rate and extent of this proteolysis and thereby determining meat tenderness (29, 30). GWAS signals in genomic regions containing CAPN and CAST genes have been reported in meat goat breeds (28), connecting these well-established biochemical pathways directly to genomic variation underlying commercially important quality traits. The genetic architecture of reproductive traits—particularly litter size and ovulation rate—in goats is governed by the TGF-*β*/BMP signaling pathway that regulates oocyte–granulosa cell communication during folliculogenesis (31). GDF9 and BMP15 are oocyte-derived paralogs of the TGF-β superfamily: they are secreted by the oocyte and signal through type-I and type-II BMP receptors on surrounding granulosa cells to regulate follicular development, granulosa cell proliferation, and ultimately ovulation quota (32). BMPR1B, which carries the well-characterized FecB mutation, acts as the primary type-I receptor for the BMP15 signaling arm of this pathway; the FecB substitution in its intracellular kinase domain reduces BMP15 pathway activity, releasing the normal suppression of follicle maturation and thereby increasing ovulation rate in heterozygous carriers (31, 32). In this framework, BMPR1B, GDF9, and BMP15 are not three independent prolificacy genes but rather interconnected nodes of the same signaling axis, with GWAS signals at each locus representing perturbations of the same folliculogenic pathway. Consistent with this interpretation, GDF9 and BMP15 variants have been associated with litter size in Youzhou dark goats (33), while BMPR1B variants are widely identified across high-prolificacy goat breeds worldwide (33, 34). Low-coverage whole-genome sequencing has also proven effective for GWAS of growth and reproductive traits in Chubao black-head goats (35). For fiber quality traits, the genetic architecture of cashmere and mohair production is rooted in the differentiation program of secondary hair follicle keratinocytes. KRT genes (encoding type-I and type-II hair keratin intermediate filaments) provide the primary structural scaffold of the fiber cortex and cuticle, while KRTAP genes encode a family of high-sulphur matrix proteins that fill the inter-filamentous spaces and cross-link keratin fibrils through extensive disulfide bonds with the cysteine residues of the keratin intermediate filaments (36). The degree of this cross-linking, determined in part by variation in KRTAP amino acid sequences, directly governs fiber diameter, mechanical strength, and crimp angle—the primary commercially relevant quality parameters in cashmere production (36). GWAS in Cashmere and Angora goats have consistently identified SNPs in multiple KRT and KRTAP family members as the key determinants of fiber diameter, yield, and crimp (14), and multiple KRTAP gene polymorphisms (including KRTAP15-1, KRTAP27-1, and KRTAP24-1) have been shown to affect cashmere fiber diameter in independent breed populations (36). In this framework, KRT and KRTAP variants do not act as isolated QTL but as perturbations of the follicular keratinocyte differentiation program that controls the biochemical composition of the fiber shaft.

These findings have not only deepened our understanding of the genetic mechanisms underlying economic traits in goats but have also provided clear targets for functional validation and molecular breeding applications.

Reproductive genomics deserves particular attention in goats because fertility, litter size, ovulation rate, age at first kidding, kidding interval, and reproductive longevity directly determine flock productivity and replacement efficiency. Compared with milk or growth traits, reproductive traits are often more difficult to improve through conventional selection because they may have low to moderate heritability, sex-limited expression, and strong environmental influence. Candidate genes involved in ovarian folliculogenesis and oocyte development, especially BMPR1B, GDF9, and BMP15, are therefore important targets for both association studies and MAS.

In practical breeding programs, MAS for major reproductive genes can be more feasible than whole-genome GS when genotyping budgets, phenotype recording, and reference population size are limited. However, MAS should be applied with caution because the effect of a reproductive mutation may differ among breeds, and favorable effects on litter size may be accompanied by management challenges such as higher kid mortality or increased nutritional demand. Therefore, reproductive markers should ideally be validated within the target breed and incorporated into a balanced selection index rather than used as single-trait selection criteria.

### 3.3 Genetic diversity, linkage disequilibrium, and breed conservation

SNP chips are powerful tools for assessing population genetic structure, genetic diversity, linkage disequilibrium (LD) patterns, and breed purity. Colli et al. (12) used the 52 K GoatSNP50 chip to construct the most comprehensive global goat genetic map to date, revealing strong partitioning of diversity across continents and providing evidence for post-domestication migration routes. Whole-genome resequencing has further illuminated the selection signatures and genetic diversity of diverse dairy goat breeds, identifying genomic regions bearing evidence of historical artificial selection (37, 38).

LD analysis, quantified by the coefficient r^2^, characterizes the extent of non-random allelic associations across the genome and directly determines the minimum SNP density required for effective GS and GWAS. In goats, LD decays substantially faster than in cattle reaching r^2^ < 0.3 within 200–500 kb in most breeds reflecting the species’ greater effective population size (Ne) and longer history of outbreeding. Berihualy et al. (6) estimated LD structure and effective population sizes in six Chinese goat populations using the 50 K panel, finding that Ne has declined markedly over recent centuries, consistent with domestication bottlenecks and selective breeding. Thomas et al. (7) conducted parallel analyses in Indian breeds, providing a basis for conservation planning for these regionally important genetic resources. These LD parameters are fundamental for designing population-optimized SNP panels and for setting minimum sample sizes for reliable GS reference populations. The rapid LD decay in goats also explains why higher-density chips (>50 K) provide meaningful accuracy gains compared to cattle, where 50 K is often sufficient.

High-density SNP markers additionally serve as precise tools for parentage verification (16), breed traceability, and detection of population admixture (12). These applications support breeding program management by ensuring pedigree accuracy, which is critical for GS, and product authentication for high-value specialty products such as cashmere and specific breed-derived cheeses (16). The conservation of endangered indigenous breeds is also facilitated by SNP-based genomic surveillance, enabling the detection of genetic erosion and guiding the selection of conservation animals to maximize retained diversity (39).

## 4 Comparison of GS and traditional breeding, and economic benefits

### 4.1 Genomic versus traditional selection: a critical comparison

GS offers fundamental advantages over traditional pedigree-based BLUP evaluation in several dimensions, while also introducing new requirements and constraints (Table 4). The most critical theoretical distinction is GS’s ability to capture Mendelian sampling variation-the random segregation of chromosomal segments that determines why full siblings differ genetically despite sharing 50% average relatedness. Traditional BLUP assigns identical within-family EBVs to sibs in the absence of progeny data; GS resolves this variation using actual genomic information. In practice, this translates to selection accuracies of r = 0.55–0.80 for dairy traits in well-resourced goat populations, compared to r = 0.30–0.55 achievable through BLUP with limited progeny records (9). A direct comparison of traditional EBVs and GEBVs in Saanen and Alpine goats confirmed the superior information content of genomic breeding values for ranking selection candidates (40). Crucially, GS enables selection of young animals, even at the embryo or yearling stage, dramatically shortening the generation interval. In dairy goats, this can reduce the time between selection decisions from four to 5 years (under progeny testing) to less than 1 year, roughly doubling the annual rate of genetic gain under equivalent selection intensities (4, 41). However, the successful implementation of GS is contingent on establishing and maintaining a large, well-phenotyped, and genotyped reference population with a genetic background representative of the selection candidates. Establishing such a population requires substantial upfront investment in phenotyping infrastructure and genotyping, constituting the primary barrier to GS adoption in small or resource-limited breeding programs (4).

**Table 4:** Comparison of genomic selection (GS) and traditional breeding methods.

Criterion	Genomic selection (GS)	Traditional breeding (BLUP-EBV)
Information source	Genome-wide SNP markers + phenotypes + pedigree (8, 11)	Phenotypic records + pedigree data (21)
Core principle	Predict GEBV using genome-wide markers; captures Mendelian sampling variation (8)	Estimate EBV via BLUP; captures only family-mean genetic value (21)
Selection accuracy	High (r = 0.55–0.80); captures within-family genetic variance not accessible to pedigree BLUP (9, 40)	Moderate; limited by heritability and data completeness; full-sib genetic merit indistinguishable without progeny data (21)
Generation interval	Shortened significantly (selection at weaning or embryo stage); 30–50% reduction possible (4, 41)	Longer; bucks require progeny testing (3–5 years for dairy traits) (21)
Novel and difficult traits	Feasible: feed efficiency, disease resistance, methane emissions, fibre quality included in selection index (3)	Difficult: sex-limited, slaughter, or expensive phenotypes require costly data collection (3)
Inbreeding management	Enables Optimum Contribution Selection (OCS) using genomic G-matrix; more accurate ΔF control per generation (4)	Relies on pedigree-based relationships; less precise inbreeding estimation in outbred populations (4)
Initial cost	High upfront (genotyping infrastructure, reference population phenotyping); liquid-phase chips progressively reducing cost (16, 17)	Lower initial cost; ongoing cost of progeny testing and phenotype recording over time (42)
Reference population dependency	Critical: large, genotyped + phenotyped reference required; accuracy declines with genetic distance from reference [4]	No genotyping required; historical pedigree data sufficient (21)
Annual genetic gain	Potentially 1.5–2 × greater than traditional methods under equivalent selection intensity, based mainly on small-ruminant simulation evidence; goat-specific economic validation remains limited (4, 42)	Baseline; well-established but limited by long generation intervals (42)

A critical but often underappreciated advantage of GS is its compatibility with Optimum Contribution Selection (OCS), which simultaneously maximizes genetic gain and constrains the rate of inbreeding increase (ΔF) at a predefined target. In traditional programs, pedigree-based OCS is limited by the imprecision of pedigree-derived relationship estimates; genomic OCS uses the G-matrix to more accurately constrain realized ΔF, preventing the long-term fitness depression associated with uncontrolled inbreeding. For goat populations with small effective sizes-particularly endangered local breeds or specialized cashmere populations-genomic OCS is an especially important tool for sustainable genetic improvement.

### 4.2 Economic benefit assessment

Despite higher initial investment, the long-term economic returns from GS are substantial and arise from multiple pathways. First, by increasing selection accuracy and shortening the generation interval, GS has the potential to accelerate the annual rate of improvement for target economic traits. However, the frequently cited 1.5- to 2-fold increase in genetic gain is derived mainly from small-ruminant simulation studies, particularly sheep breeding programs, and should therefore be interpreted as indicative rather than direct goat-specific economic evidence (42). Comprehensive cost–benefit analyses for goat GS remain limited. Current goat-specific evidence more strongly supports practical benefits such as earlier selection of young bucks, reduced reliance on progeny testing, improved evaluation of sex-limited dairy traits, and more accurate management of inbreeding and mating plans. Second, GS enables the accurate evaluation of young bucks without the need for expensive and time-consuming progeny testing, eliminating several years of feeding and management costs for candidate sires (41). Third, the inclusion of economically important but difficult-to-measure traits such as feed conversion efficiency, disease resistance, and environmental methane emissions becomes tractable under GS, enabling breeding programs to target sustainability-relevant traits that would be prohibitively costly to address through traditional selection (3). Fourth, more precise genomic information enables optimized mating decisions that avoid pairing individuals who carry complementary deleterious recessive alleles or that concentrate genetic merit in specific market-relevant trait combinations. A simulation by Shumbusho et al. (42) demonstrated that even though accounting for genotyping costs, GS provides markedly superior long-term return on investment compared to traditional schemes in small ruminants, particularly when multiple traits are included in the selection index. As liquid-phase genotyping panels reduce per-sample costs to levels comparable with parentage testing, the economic threshold for GS adoption in commercial goat programs is falling rapidly.

The economic threshold for adopting GS in goats is also highly context-dependent. Intensive dairy goat programs with established milk recording and pedigree databases are more likely to recover genotyping costs through improved selection accuracy and shorter generation intervals. In contrast, smallholder or indigenous goat systems may first benefit from lower-cost approaches, including parentage testing, candidate-gene selection, MAS for validated major genes, or low-density panels combined with imputation. As liquid-phase SNP panels reduce genotyping costs, goat-specific economic evaluations that include breed structure, recording cost, market value of target traits, and expected genetic gain are urgently needed.

## 5 Future directions and challenges

### 5.1 Reference population construction and optimization

The size and genetic composition of the reference population are the primary determinants of GEBV accuracy. Future strategies should focus on establishing multi-breed and multi-country joint reference populations that pool genotyped and phenotyped animals across national programs, an approach demonstrated to be effective in the French multi-breed dairy goat scheme (41). The ssGBLUP framework, which seamlessly integrates historical pedigree-based EBVs with genomic data from genotyped animals, maximizes the utility of legacy phenotypic records that pre-date genotyping (4). Dynamic algorithms for optimizing reference population composition selecting the most informative individuals based on genetic relationship to selection candidates will further improve resource efficiency.

### 5.2 Global representation and indigenous adaptation

A major limitation of current goat genomics is the uneven global representation of studied populations. Many SNP chip validation, GWAS, and GS studies are concentrated in European, North American, and Chinese dairy or fiber breeds, whereas African and South Asian goats remain underrepresented despite their large population sizes, high genetic diversity, and importance in smallholder systems. These regions contain numerous indigenous breeds adapted to heat, drought, seasonal feed scarcity, parasites, disease pressure, and low-input management. Underrepresentation of these populations may reduce the transferability of SNP panels, imputation reference sets, and genomic prediction models.

Future goat genomic programs should therefore prioritize African, South Asian, and other locally adapted populations in chip validation, reference population construction, and GWAS. Traits related to climate resilience, disease resistance, reproductive efficiency, kid survival, and feed efficiency under harsh environments should receive greater attention alongside milk, meat, and fiber production. Multi-country reference populations and locally optimized low-cost genotyping panels may be especially valuable for these regions, provided that benefit-sharing, breed conservation, and smallholder breeding objectives are incorporated into program design.

### 5.3 Genotyping strategies and cost reduction

Finding the optimal balance between cost and accuracy remains a central challenge for commercial GS adoption. Low-density chip plus imputation strategies offer a promising path: selection candidates are genotyped with low-cost panels (5 K–15 K SNPs) and imputed to higher density, while key ancestors and reference animals are genotyped at higher density (18). Custom liquid-phase capture panels designed around population-specific LD structure and breed-relevant genomic regions further reduce costs without sacrificing accuracy for target traits (16, 17). As sequencing costs continue to decline, low-coverage whole-genome sequencing (lcWGS) where reads at 0.5–2 × depth are imputed to full genotype calls is emerging as an alternative to array-based genotyping that avoids the design constraints of fixed-marker chips (35).

### 5.4 Multi-omics integration and complex trait dissection

Many important economic traits in goats are complex polygenic phenotypes influenced by thousands of loci of small effect, making their genetic dissection challenging. Whole-genome sequencing (WGS) data provides a near-complete catalog of sequence variants and enables the discovery of structural variants (SVs) including copy number variants and inversions that SNP chips do not capture but may explain substantial proportions of trait variance (5). Integrating genomics with transcriptomics (RNA-seq), proteomics, and metabolomics in a multi-omics framework provides a more complete mechanistic understanding of the biological basis of complex traits, including the identification of regulatory variants that act through gene expression changes rather than protein sequence alterations (43, 44). Long-read sequencing platforms (PacBio HiFi, Oxford Nanopore) are increasingly being applied to resolve complex genomic regions including the casein gene cluster and keratin gene arrays that are poorly characterized by short-read data, potentially uncovering novel functional variants of large effect.

### 5.5 Artificial intelligence and machine learning in GS

The application of artificial intelligence (AI) and machine learning (ML) methods to genomic prediction represents a rapidly expanding frontier. Deep learning architectures including convolutional neural networks (CNNs) and recurrent neural networks (RNNs) can capture complex non-linear interactions among markers and between genotype and environment that are not accommodated by linear models such as GBLUP. These approaches have shown promise for traits with non-additive genetic architecture, including disease resistance and reproductive fitness. Ensemble methods that combine predictions from multiple GS models linear mixed models, Bayesian approaches, and neural networks are also being explored to improve robustness across trait types and population structures. While the computational demands and interpretability of deep learning models remain challenges, ongoing advances in explainable AI (XAI) are beginning to address these limitations, and the integration of AI into routine genomic evaluation pipelines is an active area of development across livestock species.

### 5.6 Climate adaptation breeding

The increasing frequency and severity of climate change impacts including heat stress, drought, and emerging disease pressures highlight the importance of incorporating climate adaptation traits into goat breeding objectives. Population genomic analyses have identified signatures of positive selection in indigenous goat breeds adapted to extreme environments, revealing candidate genes associated with thermotolerance, water balance, and oxidative stress response (38, 39). Incorporating these climate-relevant traits into multi-trait GS models, along with the development of SNP panels enriched for markers in adaptation-associated genomic regions, represents an emerging strategy for future-proofing goat genetic improvement programs.

### 5.7 Gene editing technologies

The maturation of CRISPR/Cas9 and related gene editing technologies offers the possibility of directly and precisely modifying the goat genome to achieve breeding objectives that would require decades of conventional selection. Potential applications include the targeted introduction of favorable alleles identified by GWAS (e.g., the FecB mutation for prolificacy, or casein protein variants for improved milk cheese-making properties) and the elimination of deleterious recessive alleles from breeding populations (45, 46). While current applications remain largely experimental, ongoing advances in delivery efficiency, off-target effect reduction, and the regulatory landscape governing gene-edited livestock are progressively reducing barriers to translational application. Ethical, regulatory, and public acceptance considerations remain important factors that will shape the trajectory of gene editing in commercial goat breeding.

## 6 Conclusion

SNP chip technology and genomic selection have become the core engines driving goat breeding into an era of precision genomics. The progressive development from the foundational 52 K array through high-density and cost-effective liquid-phase platforms has made genome-wide genetic evaluation increasingly accessible. GS has demonstrated consistent ability to accelerate genetic progress for dairy, meat, cashmere, and reproductive traits across multiple countries and breeds, while GWAS continues to expand the catalog of genes and variants underlying economically important phenotypes. Looking forward, the integration of genotype imputation, multi-omics data, AI-assisted prediction, climate adaptation breeding, and gene editing technologies will collectively define the next generation of goat genomic improvement. Overcoming the remaining barriers particularly the construction of large, representative reference populations and the reduction of genotyping costs for small-scale programs will be essential for translating the demonstrated potential of these technologies into widespread practical benefit for goat producers and consumers worldwide.

Importantly, genomic technologies should not be viewed as a single replacement for all existing breeding approaches. Candidate-gene selection and MAS remain useful for validated major genes and resource-limited systems, whereas GS is most powerful for polygenic traits when supported by large reference populations and high-quality phenotypes. The future impact of SNP chips in goats will therefore depend not only on marker density, but also on cost-effective platform choice, population-specific validation, global representation of indigenous breeds, and integration of genomic information into realistic breeding objectives.
